# Vegans, Vegetarians and Pescatarians Are at Risk of Iodine Deficiency in Norway

**DOI:** 10.3390/nu12113555

**Published:** 2020-11-20

**Authors:** Synne Groufh-Jacobsen, Sonja Y. Hess, Inger Aakre, Elin Lovise Folven Gjengedal, Kristina Blandhoel Pettersen, Sigrun Henjum

**Affiliations:** 1 Department of Nursing and Health Promotion, Faculty of Health Science, Oslo Metropolitan University, 0130 Oslo, Norway; Kristina.blandhoel@gmail.com (K.B.P.); shenjum@oslomet.no (S.H.); 2 Department of Nutrition, Institute for Global Nutrition, University of California Davis, Davis, CA 95616, USA; syhess@ucdavis.edu; 3 Department of Seafood and Nutrition, Institute of Marine Research, 5817 Bergen, Norway; inger.aakre@hi.no; 4 Faculty of Environmental Sciences and Natural Resource Management, Norwegian University of Life Sciences, 1432 Aas, Norway; elin.gjengedal@nmbu.no

**Keywords:** iodine intake, iodine knowledge, micronutrients, pescatarians, plant-based diet, supplementation, urinary iodine concentration, vegans, vegetarians

## Abstract

Low iodine intakes have been documented in different population groups in Norway. We aimed to assess iodine status, dietary intake, supplement and macroalgae use, and iodine knowledge in vegans, vegetarians and pescatarians. In this study, 115 vegans, 55 vegetarians and 35 pescatarians from the Oslo region of Norway, aged 18–60 years, participated. A spot urine sample was collected along with a dietary assessment of iodine intake, supplement and macroalgae use. The median urinary iodine concentration (MUIC) in vegans was 43 µg/L (moderate iodine deficiency), in vegetarians 67 µg/L and in pescatarians 96 µg/L (mild iodine deficiency). In multiple linear regression analysis, use of iodine supplements was one of the strongest predictors of UIC. About half of the participants had median 24-h iodine intakes below estimated average requirement (EAR) of 100 µg/day. Fifty percent had low knowledge score, while 27% had very low knowledge score. Vegans, vegetarians and possibly pescatarians in Norway, are unable to reach the recommended iodine intake merely from food and are dependent on iodine supplements. There is an urgent need for dietary guidance targeting vegans, vegetarians and pescatarians to avoid inadequate iodine intake in non-supplement users, as well as avoiding excess iodine intake in macroalgae users.

## 1. Introduction

Plant-based diets, especially veganism, are increasing all over the world [1]. In Norway, 2–4% reported to be adhering to a vegan or vegetarian dietary practice in 2004 [2], today the number has most likely increased further. According to the Danish Vegan Society, 2.5% of the Danish population is vegan or vegetarian [3]. In Sweden the number of vegans and vegetarians increased from 3 to 10% between 2007 and 2014 [4]. 

Although changing from an omnivorous to a vegetarian or vegan dietary practice has been associated with several health benefits including a reduced risk of coronary heart disease [5], these diets may also be associated with an increased risk of micronutrient deficiencies [6,7]. Vegetarians omit meat and exclude milk, fish or eggs in varying degree and pescatarians omit meat but include fish and exclude milk or eggs in varying degree. Vegans consume plant-based foods and omit all types of animal-products. Thus, people adhering to a vegetarian, pescatarian or vegan diet may risk nutritional deficiencies. In Norway, the main dietary sources of iodine are milk, due to mandatory fortification of the cow’s fodder, seafood and eggs [8,9,10]. In Norway, the iodine fortification of table salt is voluntary and the permitted level of iodine is only 5 µg per gram [8], thus table salt is considered a negligible iodine source in the Norwegian diet. People who exclude or restrict intakes of milk, seafood and eggs may be susceptible to iodine deficiency in Norway.

Iodine is a trace element which is essential for the production of the thyroid hormones, therefore adequate iodine intake is important to avoid thyroid dysfunction and maintain normal physiological functions of the body [11]. Iodine deficiency has re-emerged as a public health problem in women of reproductive age in the USA, Australia, and Europe, including Norway, which has been corroborated in a number of studies lately [12,13,14,15]. Vegans and vegetarians have also been identified as a group with increased risk of iodine deficiency in Europe and the US [16,17,18,19,20]. In Norway, data on iodine intake and status in vegans, vegetarians and pescatarians are limited. Insufficient iodine status and inadequate iodine intake in a small group of vegans (*n* = 19) and ovo-lacto vegetarians (*n* = 25) was found in 2014–2015 [19] and in a small subsample (*n* = 36) among young women with vegetarian dietary practice [21]. 

In vegan diets, iodine-containing supplements and macroalgae remain the main source of iodine. However, the iodine concentration in macroalgae may vary considerably within and between species and some may also contain toxic amounts [22]. Thus, macroalgae users may be at risk of excessive iodine intake. A newly published summary regarding dietary habits in vegans and vegetarians concluded that vegans and vegetarians with no use of iodine-containing supplements or macroalgae are more susceptible to iodine deficiency and inadequate iodine intake compared to people following less restrictive diets [23]. In this study, we aimed to evaluate iodine status, dietary intake of iodine, supplement use, macroalgae use and iodine knowledge of Norwegian vegans, vegetarians and pescatarians. 

## 2. Materials and Methods 

### 2.1. Subjects

From September to November 2019, we recruited 205 participants, 115 vegans, 55 vegetarians and 35 pescatarians, from the Oslo and Akershus area in Norway through convenience sampling and snowball sampling. The participants were recruited through social media in closed Facebook groups and in online vegan and vegetarian forums. Information about the study purpose and participation were shared on the Oslo Metropolitan University website and on a website for health professionals interested in plant-based diets (HEPLA). Inclusion criteria were: (1) having a strict vegan, vegetarian or pescatarian diet for six months or more; and (2) participants must be 18 years or older. Participants who reported use of thyroid medication or consumption of meat-based products were excluded. After inclusion, participants answered an electronic questionnaire, assessing background information (age, height and weight, marital status, level of education, smoking habits, country of birth, language, etc.). Participants were asked whether milk/milk products, cheese, fish, eggs, meat/meat products or poultry were consumed, never, rarely, regularly or often. The participants were categorized as vegan if all options were reported as never and as vegetarian if intake of milk/milk products, cheese and/or eggs were reported, and the participants were categorized as pescatarian if intake of fish was reported. 

### 2.2. Study Participation 

**Figure 1:**
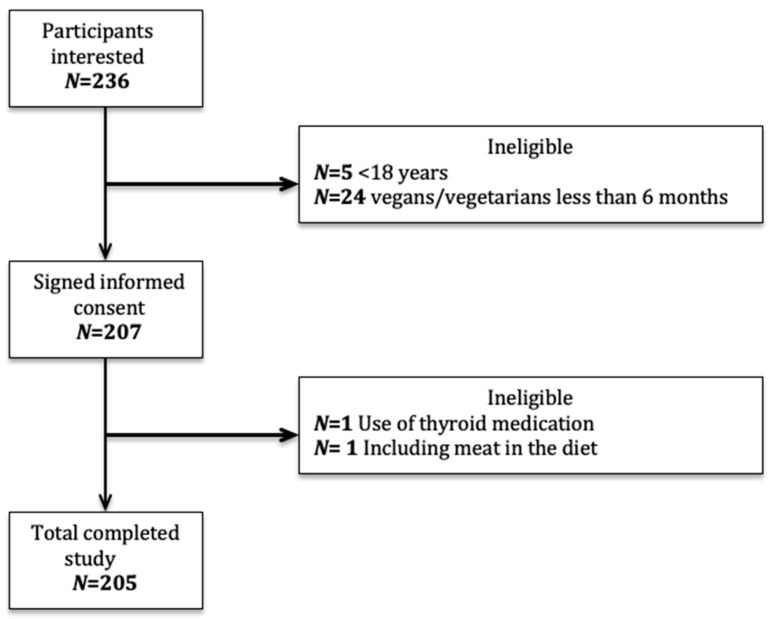
Flow chart of the recruitment.

Of the 236 individuals who expressed interest in participation (Figure 1) 29 did not meet the inclusion criteria (Figure 1). An additional two participants were excluded from analysis because of thyroid medication use and occasional meat consumption. 

### 2.3. Determination of Urinary Iodine Concentration 

All participants (*n* = 205) provided one non-fasting spot urine sample each in the morning after conducting a 24-h dietary recall. The participants were instructed to collect the spot urine sample in a labeled 100 mL Vacuette^®^ urine breaker (Greiner Bio-One, Kremsmünster, Austria). A subsample of urine was immediately withdrawn from the breaker into a 9.5 mL Vacuette^®^ urine tube (Greiner Bio-One, Kremsmünster, Austria) by a trained study staff. The urine samples were stored at 2–4 °C before freezing at −80 °C until analyses. The determination of UIC was performed at the Norwegian University of Life Science, Faculty of Environmental Science and Natural Resource Management. The frozen urine samples were thawed and subsequently subjected to an alkaline dilution. An aliquot of 1 mL of urine was transferred into 15 mL pp centrifuge tubes (Sarstedt, Nümbrecht, Germany) by means of 100–5000 µL electronic pipette (Biohit, Helsinki, Finland) and diluted to 10 mL adding an alkaline solution (BENT), containing 4% (weight (w)/volume (V) 1-Butanol, 0.1% (*w/V*) H_4_EDTA, 2% (*w/V*) NH_4_OH, and 0.1% (*w/v*) TritonTM X-100. Method blank samples and samples of standard reference material (SRM) were prepared following the same procedure with respect to alkaline dilution as the urine samples. Reagent of analytical grade or better and deionized water (>18 MΩ) were used throughout. The samples were analyzed for iodine concentration using an Agilent 8800 ICP-QQQ (Triple Quadruple Inductively Coupled Plasma Mass Spectrometer, Agilent Technologies, Hachioji, Japan) using oxygen reaction mode. The concentration of iodine in urine was determined on mass 127. ^129^I was used for correction of non-spectral interferences. The limit of detection (LOD) was 0.3 µg/L and limits of quantification (LOQ) was 0.92 µg/L. LOD and LOQ were calculated by multiplying the standard deviation of the method blank samples (*n* = 5) by three and ten, respectively. To check for method accuracy, standard reference material (SRM) of urine was analyzed. Our data were within the recommended values issued for the SeronormTM Trace Elements Urine L-1 and SeronormTM Trace Elements Urine L-2. The method repeatability was 2.2% with respect to the determination of urinary iodine concentration.

### 2.4. Assessment and Calculation of Iodine Intake from Foods

We assessed short-term intake of iodine, where time and intakes of all foods, drinks and supplements consumed during the past 24-h were assessed. The dietary interviews were conducted in person at OsloMet. Subsequently, the participants filled out an electronic FFQ to assess habitual dietary intake of iodine, consisting of 32 questions regarding their average intake of selected foods/food groups and supplements over the past four weeks based on a previously validated questionnaire used on women of fertile age and iodine status [21]. Changes were made to adapt the questionnaire to vegans’, vegetarians’ and pescatarians’ diet by adding several plant-based alternatives such as legumes, plant-based milk/yoghurt (oats, rice, soy, almond and coconut), vegan cheese and meat substitutes (soy products, tofu and tempeh). The FFQ included questions with seven frequency options ranging from “rarely/never”, “less frequently than weekly”, “1–3 times per week”, “4–6 times per week”, “1–2 times per day”, “3–4 times a day” to “5 or more times a day”. For the calculation of iodine, the reported frequency was converted from intakes the past four weeks to daily intakes by dividing the times per week with 7 days. For reported frequencies covering a range, e.g., 1–3 times per week, the middle frequency was used (2 times/week = 2/7 = 0.29 times/day). For calculation of habitual iodine intake (FFQ), the daily consumption frequency for all food items were multiplied by standard portions used in a previous study for iodine calculation [21] and the iodine concentration for each food item/dish from the updated Norwegian Food Composition Table 2019 [24]. An average value was calculated for food groups with different iodine values, such as nuts (peanuts, walnuts, etc.), lean fish (cod, saithe, etc.), sushi, cake, vegetables, etc. For the calculation of the 24-h iodine intake, we multiplied the reported daily intake with the iodine concentration applicable for each food item [24]. 

### 2.5. Assessment and Calculation of Iodine Intake from Supplements and Macroalgae

Supplement use was assessed both by 24-h and habitual intake (FFQ). The participants reported the name of the supplement, brand and amount used during the previous 24-h. By habitual intake, supplement consumption was reported as frequency per week (e.g., if a supplement contained 150 µg, and if taken 4 times a week, the contribution was estimated to be (150 µg × 4/7) 86 µg/day) by habitual use. The level of iodine concentration in macroalgae is not available in the Norwegian Food Composition table, except for Laver, and the iodine concentration may vary considerably within the same type of macroalgae [22,25,26]. To quantify the iodine intake from macroalgae, type of macroalgae used and self-reported amount (gram) were multiplied by mean iodine concentration in each type of macroalgae, which were based on reported iodine concentration in previous studies [20,22,23,24]. The types of macroalgae reported as used in this study were Sugar kelp *(Saccharina latissima)*, Bladder wrack (*Fucus vesiculosus*)*,* Wakame (*Undaria pinnatifida*), Kombu (*Laminaria japonica* and *Saccharina japonica*), Dulse (*Palmaria palmata*) and Laver (*Porphyra spp*).

**Figure 2:**
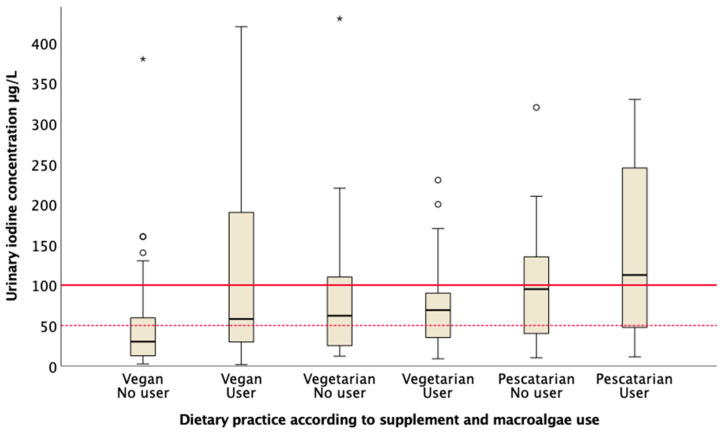
Urinary iodine concentration (µg/L) is presented according to vegan, vegetarian and pescatarian dietary practice according to use of iodine supplement reported by 24-h intake and reported habitual use of macroalgae. Vegans no user, *n* = 43; Vegan user, *n* = 72; Vegetarian no user, *n* = 26; Vegetarian user, *n* = 29; Pescatarian no user, *n* = 23 Pescatarian user, *n* = 12. Box plot details: The horizontal black lines indicate the median; the boxes indicate the interquartile range (IQR) (25th percentile to 75th percentile); the whiskers represent observations within 1.5 times the IQR. Outliers 1.5 times away from the IQR are marked as circles. Extreme outliers are marked as * and extreme outliers above 450 µg/L are excluded from the figure. The marked red line shows recommended cut-off for sufficient status in adults (100 µg/L), and the red dotted line shows cut-off for moderate iodine deficiency (50 µg/L).

To classify supplement users and non-supplement users according to UIC (Figure 2), the reported use of iodine supplements by 24-h intake was used (yes/no), combined with the reported habitual use of macroalgae (yes/no), due to lack of information of exact time of last consumption. 

### 2.6. Calculation of Iodine Knowledge Score 

A previous validated questionnaire with six questions was used to assess and calculate the iodine knowledge among the study participants [21,27]. The questionnaire had multiple answers to be able to detect people randomly answering from those knowing the answer. Correct answers generated 2 points, while correctly identified false answer generated 1 point, and incorrect answer gave 0 point. People answering multiple answer combined with “don’t know” generated 0 points. The knowledge score was divided into four groups ranging from <6–26, very low knowledge score (<6 points), low knowledge score (6–11 points), medium knowledge score (12–19 points) and high knowledge score (20–26 points).

### 2.7. Ethical Approval

Written consent was obtained from all the participants before the study start. The study had clearance from the Regional Committee for Medical and Health Research Ethics, 2019/653/REC South East and the Norwegian Center for Research Data /NSD/101332.

### 2.8. Definitions of Iodine Status and Recommended Iodine Intake 

In this study, we applied the WHO’s epidemiological criteria for assessment and evaluation of iodine status in adults [28]. Iodine status was evaluated using following median cut-off’s at population level: moderate iodine deficiency (20–49 µg/L), mild iodine deficiency (50–99 µg/L), adequate iodine nutrition (100–199 µg/L), above iodine requirements (200–299 µg/L) and excessive iodine (>300 µg/L). In addition, the WHO recommends that no more than 20% of a population should have UIC < 50 µg/L [28]. The Institute of Medicine has established an estimated average requirement (EAR) level to meet the requirement of half of the healthy individuals in a group for adults [29], while the recommended daily intake (RDI) of iodine is the level considered to meet the requirement of nearly all healthy individuals [28]. In Norway the RDI (150 µg/day) and EAR (100 µg/day) is based on the Nordic Nutritional Recommendations (NNR) [30]. As suggested by Allen, et al. we used the EAR to evaluate the adequacy of iodine intake [31]. The tolerable upper intake level (UL) is set to 600 µg/day, which is the level of daily nutrient intake that is likely to pose no risk of adverse effect in almost all individuals, thus having habitual intake above UL may impose risk of iodine excess [29]. 

### 2.9. Statistics

IBM SPSS version 25 and 27 (IBM Corp., Armonk, NY, USA) was used for statistical analysis. Normality of the data was tested using the Shapiro-Wilk test, Q–Q plots, and histograms. Normally distributed data were presented as mean ± standard deviation (SD) and non-normally distributed data as median and the 25th and 75th percentiles (p25, p75). Spearman’s correlation (r_s_) was used to evaluate the association between continuous non-parametric variables as supplement use with UIC; habitual intake of fish with UIC; iodine knowledge score with dietary intake, dietary practice with UIC, and macroalgae use with UIC. To evaluate the strength of the correlations, correlation coefficients <3 were considered as weak, correlations between 3–5 as moderate, and >5 as strong [32]. A Mann-Whitney-U-Test was used to test difference in UIC within the different dietary practices according to supplement use. Kruskal Wallis test was used to test difference in UIC between vegans, vegetarians and pescatarians. Chi-square test was used to test for difference with categorical variables. The significant level used in these tests were *p*-value < 0.05. Multiple linear regressions were used to assess the association between UIC with different independent variables. Prior to multiple regression analysis, simple regressions were performed to examine covariates (gender, smoking, pregnancy, lactation, educational level, duration of dietary practice, dietary intake, supplement use, macroalgae use and dietary practice). Independent variables that were significantly associated (*p* < 0.05) with the dependent variable (UIC) were included in a crude multiple regression model (macroalgae use, smoking, duration of dietary practice, dietary practice, 24-h iodine supplement use and education level). Variables were excluded if not significant (*p* < 0.05). In the final model, only significant variables were retained. The dependent variable UIC was skewed, and log-10 transformed prior to regression analysis. Analysis of the residuals was performed to examine the fit of the model. 

## 3. Results

Table 1 below describes the background characteristics of the dietary groups. 

### 3.1. Urinary Iodine Concentration

Median UIC (25th percentile, 75th percentile) (MUIC) in vegans was 43 (21,120) µg/L, vegetarians 67 (34, 111) µg/L and pescatarians 96 (44, 161) µg/L. According to the WHO [28], vegans were moderately iodine deficient (MUIC < 50 µg/L), while vegetarians and pescatarians were mildly iodine deficient (MUIC < 100 µg/L). Vegetarians and pescatarians had higher UIC compared to vegans, *p* = 0.030, whereas no statistical difference was found in UIC between vegetarians and pescatarians (*p* = 0.086). 

Vegans with no use of iodine supplements and no consumption of macroalgae had a lower MUIC of 31 (13, 67) µg/L compared to vegan supplement-or macroalgae users of 59 (31, 191) µg/L, *p* = 0.002 (Figure 2). There was no difference between MUIC in vegetarians with no use of iodine supplements and no use of macroalgae compared to vegetarians’ supplement-or macroalgae users, 63 (25, 116) µg/L and 70 (36, 106) µg/L, respectively, *p* = 0.742. Likewise, no difference was found in MUIC between pescatarians with no use of iodine supplements and no use of macroalgae compared to pescatarians supplement or macroalgae users, 96 (38, 141) µg/L and 114 (47, 289) µg/L, respectively, *p* = 0.424 (Figure 2).

### 3.2. Dietary Intake of Iodine from Food and Supplements 

The calculated iodine intake from foods and supplements reported in the 24-h recall and by habitual intake based on FFQ in vegans, vegetarians and pescatarians are presented in Table 2, Tables S1–S3. About half of the vegans (54%), vegetarians (51%) and pescatarians (46%) had total 24-h intakes below EAR of 100 µg/day, which may increase the risk of iodine deficiency. Intake above upper intakes level (UL) of 600 µg/day was reported in one participant in the pescatarian group. By total habitual intake, 32% of the vegans, 46% of the vegetarians and 66% of the pescatarians had intakes below EAR of 100 µg/day. Simultaneously, 18% of the vegans, 16% of the vegetarians and 9% of the pescatarians had iodine intakes above UL (600 µg/day) in macroalgae users, intake above UL habitually may impose risk of excess iodine.

### 3.3. Iodine Knowledge Score and Associations with UIC and Iodine Intake

The iodine knowledge scores among the vegans, vegetarians and pescatarians are presented in Table 3. Total iodine knowledge score differed between the dietary groups (Table 3). 

The majority (68%, *n* = 139/195) of the vegans, vegetarians and pescatarians reported to have heard about iodine and knew what iodine was, while 27% (*n* = 55/195) reported to have heard about iodine but did not remember what it was. The participants were also asked whether they believed they were getting enough iodine through their diet. Many of the participants were unsure whether they achieved the recommended iodine intake (42%, *n* = 85/195), 25% (*n* = 52/195) assumed not have an inadequate intake of iodine, and a third (28%, *n* = 58/195) of the participants assumed to have an adequate iodine intake. Total iodine knowledge score (*n* = 195) was found to be positively correlated with total habitual intake (r_s_ = 0.286, *p* ≤ 0.001), although this correlation was weak. 

### 3.4. Predictors for Urinary Iodine Concentration 

Predictors of UIC found in multiple linear regression models are presented in Table 4. Higher education (>12 years) and iodine supplement use the past 24-h were found to be associated with increased UIC, and vegan dietary practice with decreased UIC. These predictors explained 7.2% of the variance in UIC. 

## 4. Discussion

To our knowledge, this is the first study in Norway to investigate iodine status, dietary iodine intake, supplement use, macroalgae consumption and iodine knowledge in a relatively large sample of vegans, vegetarians and pescatarians. 

Among vegans, vegetarians and pescatarians in Oslo, Norway, iodine intake is below the EAR of 100 µg/day by NNR [30]. Moreover, MUIC suggests moderate iodine deficiency among vegans (MUIC <50 µg/L) and mild iodine deficiency among vegetarians (MUIC <100 µg/L) and pescatarians (MUIC <100 µg/L). Pescatarians had higher MUIC compared to vegetarians. Pescatarians who consume lean fish or mixed fish products are expected to have increased UIC compared to vegans and vegetarians. However, pescatarians who mainly consume fatty fish low in iodine are at risk of inadequate iodine intake from foods. The finding of increased MUIC in the pescatarian group in our study is mainly explained by the 24-h supplement use, as half of the participants in the pescatarian group reported a 24-h intake of iodine supplements, and only two participants reported intake of lean fish and five participants reported intake of mixed-fish products by 24-h intake, the rest of the pecatarians reported intake of fatty fish. 

The WHO recommends that the proportion with UIC <50 µg/L should be <20% of the population [28]. In our study, 55% of the vegans, 38% of the vegetarians and 28% of the pescatarians had UIC <50 µg/L. This confirms that vegans, vegetarians and pescatarians who are excluding milk or eggs to be a group in risk of iodine deficiency in Norway [28], especially those not consuming iodine supplements or macroalgae. As we found higher MUIC in vegans, vegetarians and pescatarians who were iodine supplement or macroalgae users, compared to none supplement or macroalgae users. The participants were unable to reach the recommended iodine intake from food sources. Inclusion of iodine supplements or macroalgae improved iodine status, however, intake of macroalgae may also lead to excess iodine intake. 

We found difference in MUIC between supplement or macroalgae users and non-users in vegans, however, not in vegetarians and pescatarians. One explanation could be smaller sample sizes in these groups and the use of single spot urine samples, that made it difficult to detect significant differences. A higher number of the pescatarians, equal to the number of vegans may have resulted in adequate iodine status in the pescatarian group. Our finding of insufficient MUIC (<100 µg/L) in these dietary groups, mainly vegans and vegetarians has been seen in other studies in Australia, the USA, Europe and in Norway [16,17,18,19,20].

Globally, iodization of the salt used in households is the main strategy to prevent iodine deficiency at population level [28]. The iodization of table salt in Norway is voluntary with only 5 µg per gram as the permitted level [22], thus Norwegians who are adhering to a strict vegan, vegetarian or pescatarian diet are dependent on iodine sources other than salt, such as cow’s milk, eggs, lean fish, iodine enriched plant-based milk, macroalgae use or iodine supplements, to have an adequate iodine intake [33]. Cow’s milk in Norway contains 16 µg/100 g due to mandatory iodization of the cow fodder; therefore cow’s milk is one of the major iodine sources in the Norwegian diet [8]. However, plant-based milk alternatives have become more popular and only few types of plant-based milk alternatives in Norway are iodine enriched. The majority of the participants in our study reported intake of plant-based milk alternatives that were not iodine enriched. Since vegans exclude all animal derived products, iodine-containing supplements and macroalgae use may be important to ensure adequate iodine intake in this particular dietary group. However, the iodine content of macroalgae is highly variable both within and between species and by different processing methods, thus macroalgae consumers may pose a risk of excessive iodine intakes by habitual use [22].

In our study, almost half of the vegans, vegetarians and pescatarians had total 24-h iodine intakes (including food, supplements and macroalgae) below EAR of 100 µg/day [28,30]. However, we did not find the 24-h dietary iodine intake in vegans, vegetarians and pescatarians to be correlated with UIC. This could probably be explained by poor compliance with supplement use/macroalgae use or use of single spot urine samples. UIC in spot samples is the recommended method by the WHO to assess iodine status, however urinary iodine excretion can vary somewhat from day to day and even within a given day. However, this variation tends to even out among populations [28]. Low iodine intakes have also been reported in vegans and vegetarians in Europe and in the US [6,7,16,17,18,19,20,21,34]. Inadequate iodine intakes may lead to dysfunction in the thyroid hormone production [28], thus women of reproductive age adhering to a strict vegan, vegetarian or pescatarian diet who do not regularly consume iodine-containing supplements are of special concern. Inadequate maternal iodine intake during pregnancy can result in irreversible neurodevelopment deficiencies in the fetal brain [35,36,37,38,39,40]. To ensure that thyroid stores are optimized throughout the pregnancy and lactation, a 50% higher iodine intake is required to meet the increasing demand during these phases [28,30]. 

In our study, we found iodine intakes above UL (600 µg/day) in 18% of the vegans, 16% of the vegetarians and 9% of the pescatarians, all of whom reported use of macroalgae. Chronic iodine intakes above UL (600 µg/day) increase the risk of iodine excess and thyroid dysfunction [41]. Findings of excessive iodine intakes in macroalgae users were in line with a previous study [19]. We also found the iodine knowledge to be rather low in this study, which corresponds to previous studies in women of fertile age in Norway [21,27,42]. Our findings of intakes below EAR (100 µg/day) and above UL (600 µg/day) in vegans, vegetarians and pescatarians, linked with the low iodine knowledge found in the present study, emphasizes the need for improved nutritional information targeting this vulnerable group. Furthermore, information regarding the risk of including high iodine macroalgae in the diet should be communicated to consumers. 

We found vegan dietary practice, 24-h iodine supplement use and educational level to predict 7% of the variance in UIC, which suggests that a large variation in UIC may be explained by other factors not captured in our data. These could be poor compliance with reported supplement use or macroalgae use or small sample sizes in the different dietary groups. Previous studies have also found that subjects using iodine-containing supplements or macroalgae were found to have an increased UIC compared to no-supplement users [19,23,43] and vegans to have decreased UIC compared to vegetarians [6,16,20,21], in line with our findings. 

A strength of this study was the sample size of over 200 participants, compared to previous conducted studies in vegans and vegetarians [19,21]. Another strength is the assessment of iodine intake by two different dietary methods including the assessment of iodine intake from iodine containing supplements and macroalgae, in addition to UIC. The main limitations were the recruitment methods; convenience sampling and snowball sampling. The participants were mainly from urban areas and might not be representative of all vegans, vegetarians and pescatarians living in other parts of Norway. 

## 5. Conclusions

Vegans, vegetarians and pescatarians in Norway are at risk of iodine deficiency and have limited knowledge of iodine. They were unable to reach recommended iodine intake merely from food sources alone. Pescatarians had higher iodine intake than the vegans and vegetarians, however still below the EAR. Further research on determination of the iodine content of common species of macroalgae are needed to be able to evaluate the iodine intake in macroalgae users. Furthermore, data on thyroid function in vegans, vegetarians and pescatarians are needed to fully understand the impact and importance or possible consequences of iodine supplement use or macro algae use when adhering to a vegan, vegetarian or pescatarian diet in Norway.

## Figures and Tables

**Table 1:** Background characteristics of people with vegan, vegetarian and pescatarian dietary practice in Norway (*n* = 205).

	Vegans ^a^	Vegetarians ^a^	Pescatarians ^a^	Total
	*n* (%)	*n* (%)	*n* (%)	*n* (%)
Participants	115	55	35	205
Gender				
Females	74 (64)	43 (78)	31 (89)	148 (72)
Males	41 (36)	12 (22)	4 (11)	57 (28)
Planning pregnancy, (2 years period)				
Yes	14 (19)	5 (12)	6 (19)	25 (17)
No	58 (78)	38 (88)	25 (81)	121 (82)
Currently pregnant	2 (3)	0	0	2 (1)
Country of origin				
Norway	96 (84)	45 (82)	29 (83)	170 (83)
Other countries	19 (16)	10 (18)	6 (17)	35 (17)
Age ^b^	31 ± 9 (18–56)	30 ± 10 (18–60)	28 ± 8 (20–52)	30 ± 9 (18–60)
Body mass index, kg/m^2 b^	23 ± 3 (17–33)	24 ± 4 (18–40)	23 ± 3 (18–32)	23 ± 3 (17–40)
Educational level				
<12 years	3 (3)	1 (2)	2 (6)	6 (3)
12 years	22 (19)	11 (20)	3 (9)	36 (18)
1–4 years university	90 (78)	43 (78)	30 (86)	163 (80)
Smoking status				
No	103 (89)	49 (89)	33 (94)	185 (90)
Yes	12 (11)	6 (11)	2 (6)	20 (10)
Duration of vegan/vegetarian diet (years) ^b^	4 ± 3 (0.11–10)	6 ± 3 (0.11–10)	5 ± 4 (0.11–10)	5 ± 3 (0.11–10)
Iodine supplement use, 24-h				
Yes	57 (49)	25 (45)	18 (51)	100 (49)
No	58 (51)	30 (55)	17 (49)	105 (51)
Iodine supplement use, habitually				
Yes	69 (60)	26 (47)	10 (29)	105 (51)
No	46 (40)	29 (53)	25 (71)	100 (49)
Consumption of macroalgae habitually				
Yes	23 (20)	8 (15)	4 (11)	35 (17)
No	92 (80)	47 (85)	31 (89)	170 (83)

^a^ Percentage within the group; ^b^ Mean values ± SD (min-max).

**Table 2:** Calculated daily iodine intake from one 24-h recall and habitual daily intake of iodine based on a food frequency questionnaire over the previous 4 weeks for vegans (*n* = 115), vegetarians (*n* = 55) and pescatarians (*n* = 35).

		Vegan ^a^	*n*	Vegetarian ^a^	*n*	Pescatarian ^a^	*n*	*p*-Value ^b^
24-h intake	Food only	19 (12, 30)	115	17 (12, 30)	55	16 (12, 29)	35	0.732
	Supplements	150 (150, 225)	57	150 (150, 150)	25	150 (150, 206)	18	0.723
	Total intake ^c^	92 (19, 171)	115	70 (17, 165)	55	123 (16, 176)	35	0.720
Habitual intake	Food only	16 (11, 21)	115	16 (11, 23)	55	20 (15, 30)	35	0.037 *
	Supplements	150 (150, 150)	69	150 (150, 150)	26	150 (150, 225)	10	0.061
	Macroalgae	865 (364, 1978)	23	843 (705, 1590)	8	375 (110, 610)	4	0.091
	Total intake ^c^	315 (19, 361)	115	305 (15, 323)	55	39 (16, 324)	35	0.157

^a^ Median (p25, p75); ^b^ Test for difference between the dietary groups- Kruskal Wallis test; * significance <0.05; ^c^ Total intake includes food and supplement use and macroalgae use; for more details on iodine intake in vegans, vegetarians and pescatarians see Supplemental Tables S1–S3.

**Table 3:** Iodine knowledge scores in vegans, vegetarians and pescatarians in Norway, ranging from 0–26 points, *n* = 195.

Iodine Knowledge Score	Vegans (*n* = 111) *n* (%)	Vegetarians (*n* = 53) *n* (%)	Pescatarians (*n* = 31) *n* (%)	*p*-Value ^a^	Total (*n* =195) *n* (%)
Very low (0–5 points)	24 (22)	14 (26)	15 (48)		53 (27)
Low (6–11 points)	55 (50)	28 (53)	14 (45)		97 (50)
Medium (12–19 points)	32 (29)	11 (21)	2 (7)		45 (23)
High (20–26 points)	0	0	0	0	0
Mean score (± SD)	8 ± 4	8 ± 4	5 ± 4	0.001	

^a^ Test for difference between the dietary groups–Kruskal Wallis test.

**Table 4:** Predictors of urinary iodine concentration in vegans, vegetarians and pescatarians (*n* = 205).

Dependent Variable	Predictor Variables	Unadjusted Coefficient (CI ^5^ 95%)	*p*-Value	Adjusted Coefficient ^6^ (CI ^5^ 95%)	*p*-Value
UIC, µg/L ^1^					
	Vegan dietary practice ^2^	−0.2 (−0.31, −0.04)	0.01	−0.2 (−0.16, −0.02)	0.01
	Iodine supplement use, last 24 h ^3^	0.2 (0.02, 0.29)	0.02	0.2 (0.01, 0.15)	0.03
	Higher Education ^4^	0.2 (0.00, 0.34)	0.01	0.1 (0.00, 0.14)	0.05

^1^ Urinary iodine concentration µg/L was LOG 10- transformed; ^2^ Vegan dietary practice (0 = vegetarian and pescatarians, 1 = vegans); ^3^ Iodine supplement use (0 = no, 1 = yes); ^4^ Higher education (0 = less than 12 years education, 1 = more than12 years education); ^5^ Confidence interval; ^6^ Adjusted for vegan dietary practice, 24-h iodine supplement use; higher education; Independent variables that were not significant or further increasing the explained variance were not included in this final model (gender, smoking, pregnancy, lactation, educational level, duration of dietary practice, dietary intake, habitual macroalgae use, habitual supplement use).

## Supplementary Materials

The following are available online at https://www.mdpi.com/2072-6643/12/11/3555/s1, Table S1: Iodine intake from foods, supplements and macroalgae by 24-h and habitual intake in vegans (*n* = 115), Table S2: Iodine intake from foods, supplements and macroalgae by 24-h and habitual intake in vegetarians (*n* = 55), Table S3: Iodine intake from foods, supplements and macroalgae by 24-h and habitual intake in pescatarians (*n* = 35).
