# Supplementary material for: Vegans, Vegetarians and Pescatarians Are at Risk of Iodine Deficiency in Norway
Source: Nutrients. 2020 Nov 20;12(11):3555. doi: 10.3390/nu12113555 (PMC7699510; doi:10.3390/nu12113555)
Supplement: Supplementary file 1 [file nutrients-12-03555-s001.pdf]

Table S1. Iodine intake from foods, supplements and macroalgae by 24-hour and habitual intake in vegans (*n*=115).

Vegans		Calculated iodine intake µg/day (Median (IQR))				P-value [a]
**24-hour intake**		All (*n*=115)	Non supplement user (*n*=58)	Supplement user (*n*=57)		
	Food only	19 (12, 30)	19 (12, 34)	19 (13, 25)		
	I Supplements only	150 (150, 225)	NA	150 (150, 225)		
	Total intake	92 (19, 171)	19 (12, 34)	171 (163, 261)		<0.001**
	% (n/N) below the EAR (100 µg/day)	54% (62/115)	100% (58/58)	7% (4/57)		
	% (n/N) above the UL (600 µg/day)	0% (0/115)	0% (0/58)	0% (0/57)		
**Habitual intake**		All (n=115)	Non supplement user (n=46)	Supplement user (n=69)	Macroalgae user (n=23)	
	Food only	16 (11, 21)	15 (11, 19)	17 (11, 22)	13 (11, 24)	
	I Supplements only	150 (150, 150)	NA	150 (150, 150)	150 (150, 150)	
	Macro-algae only	865 (364, 1978)	1300 (705, 2289) (n=9)	705 (266, 1925) (n=14)	865 (364, 1978)	
	Total intake	315 (19, 361)	15 (11, 19)	321 (314, 440)	1011 (639, 1982)	<0.001**
	% (n/N) below the EAR (100 µg/day)	32% (37/115)	80% (37/46)	0% (0/69)	0% (0/23)	
	%(n/N) above the UL (600 µg/day)	18% (21/115)	17% (8/46)	19% (13/69)	79% (18/23)	

[a] Kruskal Wallis test was used to test difference in total intake between non-supplement users, supplement users and macroalgae users, significant p-values <0.001 are marked as **.

Table S2. Iodine intake from foods, supplements and macroalgae by 24-hour and habitual intake in vegetarians (*n*=55).

**Vegetarians**		**Calculated iodine intake µg/day (Median (IQR))**				**P-value** [a]
**24-hour intake**		All (n=55)	Non supplement user (n=30)	Supplement user (n=25)		
	Food only	17 (12, 30)	19 (12, 34)	15 (12, 25)		
	I Supplements only	150 (150, 150)	NA	150 (150, 150)		
	Total intake	70 (17, 165)	19 (12, 34)	167 (162, 181)		<0.001**
	% (n/N) below the EAR (100 µg/day)	51% (28/55)	93% (28/30)	0% (0/25)		
	% (n/N) above the UL (600 µg/day)	0% (0/55)	0% (0/30)	0% (0/25)		
**Habitual intake**		All (n=55)	Non supplement user (n=29)	Supplement user (n=26)	Macroalgae user (n=8)	
	Food only	16 (11, 23)	15 (11, 23)	16 (12, 23)	21 (8, 26)	
	I Supplements only	150 (150, 150)	NA	150 (150, 150)	150 (150, 750)	
	Macro-algae only	843 (705, 1590)	1176 (274, 2510) **(n=4)**	843 (705, 1303) **(n=4)**	843 (705, 1590)	
	Total intake	305 (15, 323)	16 (12, 33)	318 (312, 553)	1480 (586, 2126)	<0.001**
	% (n/N) below the EAR (100 µg/day)	46% (25/55)	86% (25/29)	0% (0/26)	0% (0/8)	
	%(n/N) above the UL (600 µg/day)	16% (9/55)	10% (3/29)	23% (6/26)	80% (6/8)	

[a] Kruskal Wallis test was used to test difference in total intake between non-supplement users, supplement users and macroalgae users, significant p-values <0.001 are marked as **.

Table S3. Iodine intake from foods, supplements and macroalgae by 24-hour and habitual intake in pescatarians (*n*=35).

**Pescatarian**		**Calculated iodine intake µg/day (Median (IQR))**				**P-value [a]**
**24-hour intake**		All (n=35)	Non supplement user (n=17)	Supplement user (n=18)		
	Food only	16 (12, 29)	16 (8, 32)	19 (13, 28)		
	I Supplements only	150, (150, 206)	NA	150, (150, 206)		
	Total intake	123 (16, 176)	16 (8, 32)	173 (163, 223)		<0.001**
	% (n/N) below the EAR (100 µg/day)	46%	94%	0%		
	% (n/N) above the UL (600 µg/day)	0% (0/35)	0% (0/17)	6% (1/18)		
**Habitual intake**		All (n=35)	Non supplement user (n=25)	Supplement user (n=10)	Macroalgae user (n=4)	
	Food only	20 (15, 30)	20 (14, 36)	21 (14, 27)	18 (16, 54)	
	I Supplements only	150, (150, 225)	NA	150, (150, 225)	150 (186, 225)	
	Macro-algae only	375 (110, 610)	570 (491, 650) **(n=2)**	160 (60, 260) **(n=2)**	376 (110, 610)	
	Total intake	39 (16, 324)	20 (14, 36)	327 (314, 540)	587 (446, 714)	0.001*
	% (n/N) below the EAR (100 µg/day)	66% (23/35)	92% (23/25)	0% (0/10)	0% (0/4)	
	%(n/N) above the UL (600 µg/day)	9% (3/35)	4% (1/25)	20% (2/10)	50% (2/4)	

[a] Kruskal Wallis test was used to test difference in total intake between non-supplement users, supplement users and macroalgae users, significance level <0.05 was used marked as * and significant p-values <0.001 as **.
